# Prospective validation of a real-time natural language processing pipeline for the extraction of elastography results from electronic health record data

**DOI:** 10.1097/HC9.0000000000001000

**Published:** 2026-07-15

**Authors:** Lale A. Ertuglu, Christina Grimes, Dameia Brewster, Cathy A. Jenkins, Qingxia Chen, Dario Giuse, Ashley Spann

**Affiliations:** 1 Department of Internal Medicine, Division of Internal Medicine, Vanderbilt University Medical Center, Nashville, Tennessee, USA; 2 HealthIT, Vanderbilt University Medical Center, Nashville, Tennessee, USA; 3 Department of Internal Medicine, Division of Gastroenterology, Hepatology, and Nutrition, Vanderbilt University Medical Center, Nashville, Tennessee, USA; 4 Department of Biostatistics, Vanderbilt University Medical Center, Nashville, Tennessee, USA; 5 Department of Biomedical Informatics, Vanderbilt University Medical Center, Nashville, Tennessee, USA

**Keywords:** cirrhosis, hepatitis, liver disease, MASLD

## INTRODUCTION

Metabolic dysfunction–associated steatotic liver disease (MASLD) affects approximately one-third of the global adult population and over 70% of people with type 2 diabetes or obesity.^1^ While MASLD usually starts with hepatic steatosis, an often asymptomatic and reversible condition, it can progress to metabolic dysfunction–associated steatohepatitis (MASH) and fibrosis.^2^

Noninvasive imaging modalities, including shear wave elastography (SWE) and magnetic resonance elastography (MRE), offer rapid and accurate assessment of liver stiffness without the need for liver biopsy and have become crucial for treatment allocation and monitoring response. Both modalities provide quantitative thresholds for liver stiffness measurement (kPa) that are widely used to exclude or indicate advanced fibrosis.^3^ MRI proton density fat fraction (PDFF) further provides the gold standard noninvasive quantification of hepatic steatosis.^3^

Despite their clinical value, liver stiffness measurements are typically stored as free text within radiology reports in the electronic health record (EHR) and are not readily available for systematic identification of patients. Acquisition of these measures by manual chart review is time-intensive and prone to human error. Natural language processing (NLP) algorithms have been successfully used for the identification of MASLD and cirrhosis in EHR free-text documentations and radiology reports,^4^^,^^5^ but their utility to capture discrete fibrosis and steatosis values from SWE and MRE reports has not been evaluated. In this study, we developed and examined the performance of a real-time NLP pipeline tailored specifically to extract elastography-derived fibrosis and steatosis values.

## METHODS

### Study design and data collection

We conducted a prospective study within the EHR system at Vanderbilt University Medical Center (VUMC), including all adult patients who underwent SWE or MRE from February 11, 2025, to April 21, 2025, within the VUMC Health System. This study was conducted in accordance with both the Declarations of Helsinki and Istanbul and approved with a waiver of informed consent for providers by the VUMC Institutional Review Board (IRB 210772).

### NLP pipeline development

An NLP pipeline was designed and integrated directly into the EHR system (Epic) at VUMC to enable real-time extraction of liver elastography data from SWE or MRE reports (Supplemental Figure S1, http://links.lww.com/HC9/C395). The NLP was designed within the concept extraction NLP tool WordCloud, a high-speed, rules-based NLP system developed at VUMC and fully integrated into our EHR system.^6^ The NLP pipeline was designed to process unstructured clinical text in real time using Unified Medical Language System (UMLS) terminology. For concept identification, key concepts included liver stiffness values, reported in kPa, and liver steatosis values, reported in PDFF. EHR embedding allowed for immediate capture of SWE and MRE reports in real time. Notifications of the results were distributed to the study team at the time of reporting and were extracted from the Word Cloud database for analysis. The complete radiology texts for both SWE and MRE studies were also extracted to a secure REDCap database for comparison with Word Cloud reports for manual review. The performance of the NLP pipeline was assessed by comparing the extracted liver stiffness (kPa) and liver steatosis (PDFF) data to manual reviews of the original SWE/MRE reports conducted by trained physicians.

### Statistical analysis

Baseline data are expressed as median and interquartile range for continuous variables, and as frequencies and percentages for categorical variables. Extraction methods were compared using sensitivity, specificity, and positive and negative predictive values, and reported with their corresponding 95% Wilson confidence intervals. Statistical analysis was carried out in R software, version 4.4.0.

## RESULTS

### Patient characteristics

A total of 415 liver elastography studies (186 MRE and 229 SWE) were performed in 411 patients during the study period. The baseline characteristics of the study population are demonstrated in Supplemental Table S1, http://links.lww.com/HC9/C395.

### Outcomes assessment

#### Performance of the NLP pipeline to capture fibrosis values

The performance of the NLP pipeline is shown in Table 1, and classification tables for performance calculations are available in Supplemental Table S2. For discrete fibrosis values, the NLP pipeline demonstrated 100% sensitivity, specificity, and positive/negative predictive accuracy in diagnostic SWE and MRE reports. In all, 5 of 415 (1.2%) studies were nondiagnostic per the radiology report, and 10 out of 415 (2.4%) studies did not include a discrete fibrosis value, which were noted as true negatives in the analysis.

**TABLE 1:** Test characteristics with 95% Wilson confidence intervals for manual versus NLP extraction of liver stiffness (kPa) and assessment

	Estimate	95% CI	Number of studies
Liver stiffness			
Shear wave elastography (kPa)			229
Sensitivity	1.00	(0.98, 1.00)	
Specificity	1.00	(0.74, 1.00)	
Positive predictive value	1.00	(0.98, 1.00)	
Negative predictive value	1.00	(0.74, 1.00)	
** **MR elastography (kPa)			186
Sensitivity	1.00	(0.98, 1.00)	
Specificity	1.00	(0.51, 1.00)	
Positive predictive value	1.00	(0.98, 1.00)	
Negative predictive value	1.00	(0.51, 1.00)	
Liver fat content			
** **MR elastography (PDFF)			186
Sensitivity	0.98	(0.95, 0.99)	

*Note:* Specificity, PPV, and NPV were not reported for MRE PDFF as there were no true negatives in the sample.

Abbreviations: CI, confidence interval; MRE, magnetic resonance elastography; NLP, natural language processing; NPV, negative predictive value; PDFF, proton density fat fraction; PPV, positive predictive value.

#### Performance of the NLP pipeline to capture steatosis values

Among 186 MRE studies, the NLP pipeline successfully identified PDFF values in 182 (97.8%) for hepatic steatosis evaluation. The NLP pipeline had a sensitivity of 98% (95%, 99%), respectively, for PDFF. Four reports, which documented a single PDFF value instead of a range, were not captured by the NLP pipeline.

## DISCUSSION

We developed and validated an NLP pipeline to accurately extract exact fibrosis values from SWE and MRE reports, as well as steatosis values from MRE reports in real time. The NLP pipeline showed excellent sensitivity.

Our findings showed that real-time NLP can be effectively incorporated into EHR, enabling more efficient identification of MASLD patients and streamlining eligibility assessment for resmetirom and glucagon-like peptide-1 receptor agonist (GLP-1ra) therapy. This NLP pipeline can be utilized to trigger EHR-embedded clinical decision support systems to prevent gaps in care and improve guideline adherence to new treatment options.

In this study, we utilized an NLP pipeline instead of large language models (LLMs) for several reasons. While LLMs may be easily customized and generalized, NLP pipelines are less computationally intensive and easier to integrate into EHRs, making them a more cost-effective option for healthcare settings. Furthermore, the structured and deterministic nature of the NLP provides greater reliability and eliminates the risk of hallucinations, a phenomenon seen with LLMs.

Our study had several limitations. First, the NLP pipeline was developed and validated in one health system, and its performance depends on the institutional EHR system and documentation practices. We utilized rule-based methods, which rely on standard use of abbreviations and nomenclature in radiology reports. Our sample size for MRE reports was smaller than SWE. Furthermore, implementation in another EHR requires a custom-built NLP.

In conclusion, EHR-embedded NLP pipelines offer excellent accuracy in real-time extraction of fibrosis and steatosis values. Real-time NLP pipelines can be used in the identification of MASLD patients, the development of clinical decision support systems, and to improve the efficiency of clinical research.

## Supplementary Material
