# Supplementary material for: Prospective validation of a real-time natural language processing pipeline for the extraction of elastography results from electronic health record data
Source: Hepatol Commun. 2026 Jul 15;10(8):e01000. doi: 10.1097/HC9.0000000000001000 (PMC13671591; doi:10.1097/HC9.0000000000001000)
Supplement: Supplementary file 1 [file hc9-10-e01000-s001.docx]

**Supplementary Materials:**

**Supplementary Table: Baseline Characteristics of Patients Included in the Study**

| **Demographic Variables** | **Overall**  **(n=415)** | **Shear Wave Elastography Patients (n=229)** | **MRI Elastography Patients (n=186)** |
| --- | --- | --- | --- |
| **Age at Visit** | 53.00 (41.00–64.00) | 56.00 (42.00–66.00) | 51.00 (40.00–62.00) |
| **BMI at Visit** | 32.10 (26.95–37.60) | 28.00 (24.00–32.00) | 37.40 (34.00–40.90) |
| **Female Gender** | 234 (56.39%) | 132 (57.64%) | 102 (54.84%) |
| **Race** |  |  |  |
| White | 234 (56.39%) | 132 (57.64%) | 102 (54.84%) |
| Black | 181 (43.61%) | 97 (42.36%) | 84 (45.16%) |
| **Pre-Existing Comorbidities** |  |  |  |
| Chronic Kidney Disease | 44 (10.60%) | 22 (9.61%) | 22 (11.83%) |
| Diabetes Mellitus | 102 (24.58%) | 48 (20.96%) | 54 (29.03%) |
| Hyperlipidemia | 177 (42.65%) | 91 (39.74%) | 86 (46.24%) |
| Hypertension | 187 (45.06%) | 89 (38.86%) | 98 (52.69%) |
| **Laboratory Data** |  |  |  |
| AST | 34.00 (25.00–47.00) | 35.00 (25.00–48.00) | 32.50 (24.00–46.00) |
| ALT | 36.00 (22.00–61.00) | 34.00 (21.00–57.00) | 37.50 (23.25–68.50) |
| Platelets | 239.00 (195.50–304.00) | 235.00 (188.00–297.00) | 254.00 (201.00–306.75) |
| HgbA1c | 5.50 (5.20– 6.30) | 5.50 (5.20– 6.15) | 5.50 (5.20– 6.70) |
| FIB-4 Score | 1.17 (0.77– 1.94) | 1.42 (0.91– 2.05) | 1.04 (0.71– 1.80) |
| LDL | 100.00 (75.50–123.50) | 95.50 (73.25–119.75) | 105.00 (77.00–125.75) |
| Triglycerides | 130.50 (87.00–176.50) | 125.50 (79.50–163.00) | 137.00 (99.50–192.50) |

**Supplementary Table 2: 2x2 Classification Tables Used for Performance Calculations.**

|  | **Shear Wave Elastography (kPa)** | | **MR Elastography (kPa)** | | **MR Elastography (PDFF)** | |
| --- | --- | --- | --- | --- | --- | --- |
|  | **True positive** | **True negative** | **True positive** | **True negative** | **True positive** | **True negative** |
| **Test positive** | 218 | 0 | 182 | 0 | 182 | 0 |
| **Test negative** | 0 | 11 | 0 | 4 | 4 | 0 |

ALT, alanine aminotransferase; AST, aspartate aminotransferase; BMI, body mass index; HgbA1c, Hemoglobin A1c; LDL, low-density lipoprotein.

Data are presented as n (%) or median and IQR.

**Supplementary Figure 1: The Framework of Proposed NLP to EHR Pipeline**
